# Robotic pancreatoduodenectomy provides better short-term outcomes as compared to its laparoscopic counterpart: a meta-analysis

**DOI:** 10.3389/fonc.2025.1568957

**Published:** 2025-06-18

**Authors:** Faying Liu, Yang Zou, Qing Chen, Tao Chen, He Xiao, Tingbing Xie, Lihe Zheng, Qi Ruan, Wang Liu

**Affiliations:** Department of General Surgery, ChengFei Hospital, Chengdu, Sichuan, China

**Keywords:** robotic pancreatoduodenectomy, laparoscopic pancreatoduodenectomy, mortality, postoperative complications, meta-analysis

## Abstract

**Objective:**

Minimally invasive pancreaticoduodenectomy is becoming more and more popular among surgeons, but whether robotic pancreatoduodenectomy (RPD) is superior to laparoscopic surgery remains controversial. The study aims to assess the available literature and compare the perioperative outcomes of RPD and laparoscopic pancreatoduodenectomy (LPD).

**Methods:**

A systematic literature search was performed in the PubMed, Cochrane Library, Embase, Web of Science databases (October 2024). Risk ratios (RRs) and mean differences (MDs) with 95% confidence intervals (CIs) were calculated.

**Results:**

The 29 studies that met inclusion criteria included 15137 PDs, out of which 8935 were LPD and 6202 were RPD. Compared with LPD, RPD has lower overall complications (RR, 0.87), conversion rates (RR, 0.47) and blood transfusion rates (RR, 0.56), shorter length of stay (MD, -0.80 days), and higher number of harvested lymph nodes (MD, 1.77). There were no significant differences observed in 90-day mortality (RR, 0.92), major complications (RR, 1.00), operative time (MD, 3.93 mins), blood loss (MD, -22.50 mL), reoperation (RR, 0.96), bile leak (RR, 0.87), postoperative pancreatic fistula (RR, 1.00), delayed gastric emptying (RR, 1.19), and R0 resection (RR, 0.99) between the groups.

**Conclusions:**

Robotic-assisted surgery for PD is safe and feasible. Compared to LPD, it offers better short-term outcomes.

## Introduction

Pancreatoduodenectomy (PD) is a challenging surgical procedure associated with high postoperative complications and mortality (1). With the advancement of surgical techniques and perioperative management, although the postoperative mortality rate of PD has been reduced to 5%, the postoperative complications is still as high as 40% (2). Postoperative complications will not only prolong hospital stay and increase hospital cost, but also affect the long-term prognosis of patients (3). Therefore, how to reduce postoperative complications is the key concern of pancreatic surgeons.

Compared with traditional open surgery, minimally invasive surgery (including laparoscopic surgery and robotic surgery) may have potential advantages in reducing postoperative complications and blood loss, and shortening hospital stay (4–6). Since Gagner et al. reported the first case of laparoscopic pancreatoduodenectomy (LPD) in 1994, LPD has been widely used in the world (7). However, laparoscopic surgery has disadvantages such as unstable camera platform, limited range of motion and two-dimensional imaging (3). The robotic surgical platform has a three-dimensional visual field of view and more flexible and precise manipulation of instruments, so it retains the advantages of minimally invasive surgery while overcoming the disadvantages of laparoscopic surgery (1, 8). Several studies have compared the effectiveness and safety of robotic and laparoscopic surgery in PD. However, whether robotic pancreatoduodenectomy (RPD) is superior to LPD remains controversial. Farah et al. ‘s (4) cohort study found that RPD significantly reduced the incidence of postoperative complications compared with LPD (51% vs. 38.9%, respectively). An international multicenter retrospective study by Emmen et al. (9), including 2,082 patients from 50 centers in 12 European countries, showed that the incidence of postoperative pancreatic leakage and delayed gastric emptying was higher in the RPD group than in the LPD group.

Therefore, in order to clarify the effectiveness and safety of robotic surgery in PD and to provide evidence-based medical evidence for surgeons when selecting surgical approaches. We comprehensively collected published evidence and conducted a meta-analysis to evaluate the potential benefits of RPD versus LPD in short-term outcomes.

## Methods

### Search strategy

This study follows the Preferred Reporting Items for Systematic Reviews and Meta-Analyses (PRISMA) (10). Two authors (Faying Liu and Yang Zou) independently conducted a comprehensive literature search using the EMBASE, Web of Science, PubMed, and Cochrane Library databases to identify studies published before October 24, 2024. The search strategy is presented in **Table 1**. In addition, we checked the reference lists of the identified articles and related reviews to further screen for eligible studies. No language restrictions were applied during the search process.

**Table 1:** Search strategy.

Database	Search strategy	Number
PubMed	((da Vinci[Title/Abstract]) OR (robot*[Title/Abstract]) OR (robot-assisted[Title/Abstract]) OR (robotic-assisted[Title/Abstract])) AND ((laparoscopy[MeSH Terms]) OR (Laparoscop*[Title/Abstract])) AND ((pancreatoduodenectomy[MeSH Terms]) OR (Pancreaticoduodenectom*[Title/Abstract]) OR (Duodenopancreatectom*[Title/Abstract]) OR (Whipple[Title/Abstract]) OR (Whipple’s procedure[Title/Abstract]) OR (Kausch-Whipple[Title/Abstract]) OR (Kausch-Whipple procedure[Title/Abstract]))	339
Embase	(Pancreatoduodenectomy OR Pancreaticoduodenectom* OR Duodenopancreatectom* OR Whipple’s procedure OR Kausch-Whipple OR Kausch-Whipple procedure).ab,kw,ti. AND (Da Vinci OR Robot* OR Robot-assisted OR Robotic-assisted).ab,kw,ti. AND (laparoscopy or Laparoscop*).ab,kw,ti.	570
Cochrane Library Trials	(((Pancreatoduodenectomy) OR (Pancreaticoduodenectom*) OR (Duodenopancreatectom*) OR (Whipple’s procedure) OR (Kausch-Whipple) OR (Kausch-Whipple procedure)):ti,ab,kw) AND (((Da Vinci) OR Robot* OR Robot-assisted OR Robotic-assisted):ti,ab,kw) AND ((laparoscopy OR Laparoscop*):ti,ab,kw)	30
Web of Science	(TS=((Da Vinci) OR (Robot*) OR (Robot-assisted) OR (Robotic-assisted))) AND (TS=((laparoscopy) OR (Laparoscop*))) AND TS=((Pancreatoduodenectomy) OR (Pancreaticoduodenectom*) OR (Duodenopancreatectom*) OR (Whipple’s procedure) OR (Kausch-Whipple) OR (Kausch-Whipple procedure))	597

### Study selection

Studies included in this meta-analysis were chosen according to the PICOS criteria:

- Patient: patients undergoing pancreatoduodenectomy;
- Intervention: robotic pancreatoduodenectomy;
- Comparison: laparoscopic pancreatoduodenectomy;
- Outcomes: assessing any of the short-term outcomes of interest. Studies focusing solely on long-term survival or those without direct comparison between RPD and LPD were excluded. Primary outcomes included 90-day mortality, overall complications, and major complications (Clavien-Dindo III-V) (9). Secondary outcomes included blood loss, length of stay, operative duration, conversion, reoperation, bile leak, postoperative pancreatic fistula (POPF), delayed gastric emptying, blood transfusion, number of harvested lymph nodes, and R0 resection. 90-day mortality was defined as any death within 90 days from surgery.The overall complications were defined as any complications and classified according to the Clavien-Dindo classification (including both surgical complications and medical complications).
- Study type: RCTs, cohort studies, and case-control studies.

The exclusion criteria were as follows: reviews, case reports, editorials, conference abstracts, letters, single-arm studies, animal studies, and repeated publications. Studies with fewer than 10 patients in each group were excluded.

### Data extraction

Data from all eligible studies were independently extracted by two investigators (Faying Liu and Yang Zou), and any disagreements were resolved by discussion with a third-party independent reviewer (Qi Ruan). The extracted data included author name, year of publication, country, study design, study population (sample size, age, body mass index, and sex), and short-term outcomes. When data of interest were unavailable, the corresponding author was contacted to obtain the necessary data.

### Quality assessment

The risk of bias in RCTs was assessed independently by two authors (Faying Liu and Yang Zou) using the Cochrane risk-of-bias tool 2 (11): (1) randomization process, (2) deviations from intended interventions, (3) missing outcome data, (4) measurement of the outcome, (5) selection of reported results, and (6) overall risk of bias. For non-RCTs, the quality assessment was conducted independently by two authors using the Newcastle-Ottawa Scale (NOS), which assigns a score on a 9-point scale. A score of ≥7 indicates high quality, and scores of 5–6 indicate moderate quality. Any discrepancies were resolved through discussion, with intervention by a third author (Qi Ruan) whenever necessary.

### Statistical analysis

The meta-analysis was performed using the Review Manager software (version 5.3). Risk ratios (RR) with corresponding 95% confidence intervals (CI) were calculated for qualitative variables and mean difference (MD) for quantitative data. The I² statistic was used to assess the degree of heterogeneity. A random-effects model was used if I² > 50%; otherwise, a fixed-effects model was employed (12). To explore the robustness of the results, we adopted the 1-study exclusion method to evaluate the impact of each study on the pooled effect size. When zero events were observed in one or both treatment groups in a trial, we excluded these studies to verify the robustness of our results. Publication bias was assessed using funnel plot for primary outcomes. Statistical significance was set at p < 0.05.

## Results

### Literature retrieval

**Figure 1:**
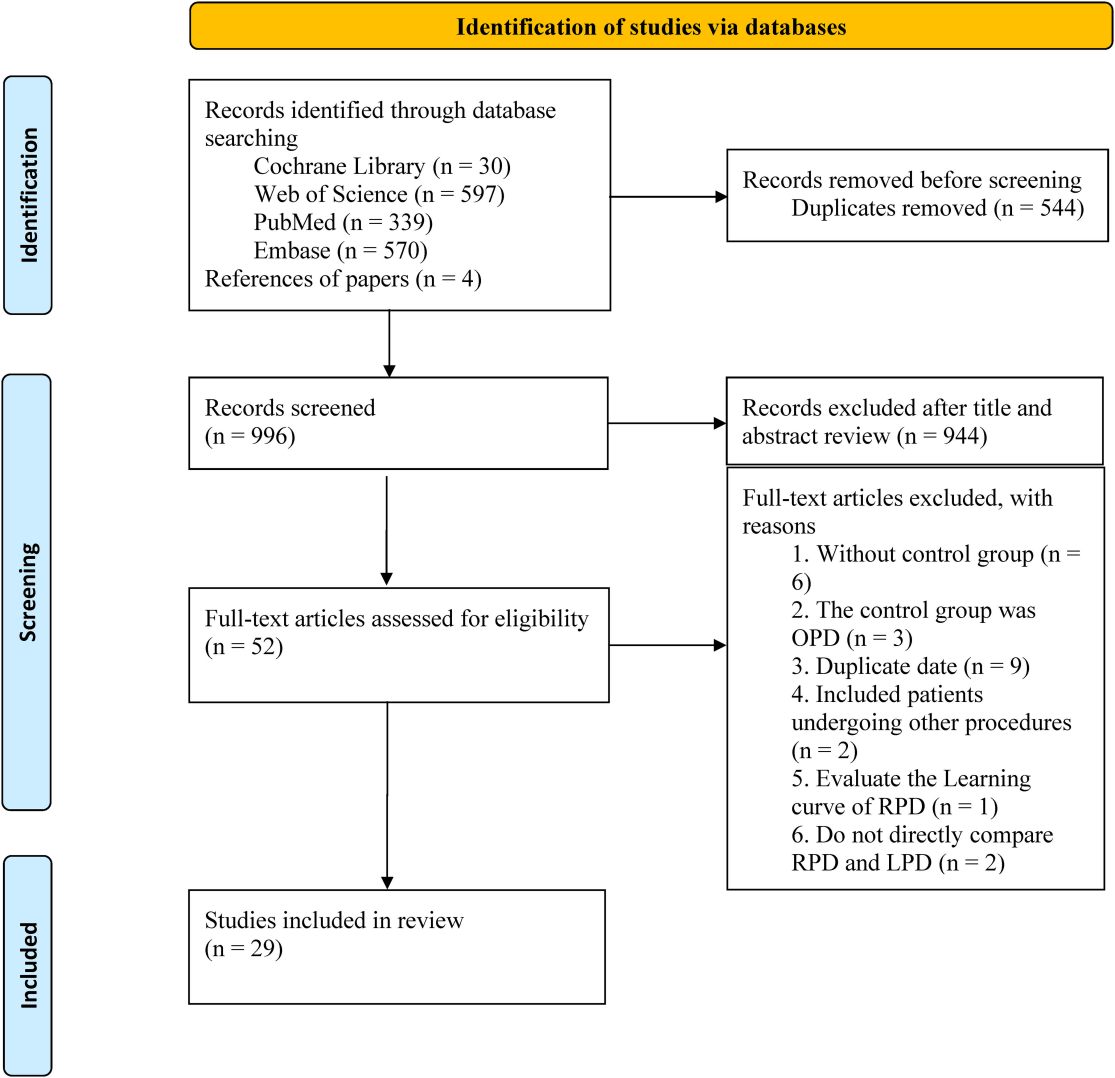
The PRISMA flowchart.

The search strategy retrieved 1540 studies, of which 544 duplicates were excluded. After reviewing titles and abstracts, 944 studies were excluded, and the full texts of the remaining 52 studies were evaluated. Finally, 29 studies (1, 4, 9, 13–38) were included in the final analysis (**Figure 1**).

### Study characteristics and quality assessment

The main characteristics of the 29 included studies are summarized in **Table 2**. The studies were published between 2016 and 2024 and included 15137 patients (RPD group: 6202 patients; LPD group: 8935 patients). Among the included studies, 27 were retrospective cohort studies and 2 were prospective cohort studies. Nine studies adopted the PSM design. The included patients were mainly from the United States, China, Korea, The Netherlands, UK, Russia, Japan, and Singapore. All studies were considered of moderate to high quality, achieving a score of ≥6 based on the NOS.

**Table 2:** Study Characteristics of the 29 included studies.

First author, year	Country	Period of study	Sample size	Male	Study design	Age	BMI	Indication for surgery	Outcomes	NOS
Liu 2016 (13)	China	2015-2016	RPD: 27 LPD: 25	RPD:14 LPD: 12	RCS	RPD: 57.16(8.56) LPD: 60.54(18.25)	RPD: NA LPD: NA	Periampullary neoplasms	Overall complications, length of stay, delayed gastric emptying, R0 resection, bile leak, blood loss, operative time, conversion, reoperation, and number of harvested lymph nodes	6/9
Goh 2018 (14)	Singapore	2014-2017	RPD: 10 LPD: 20	RPD:5 LPD: 16	RCS	RPD: 70(53-78) LPD: 62.5(24-79)	RPD: 21.3(18-27.6) LPD: 20.6(14-26)	Periampullary tumours	90-day mortality, overall complications, major complications, blood loss, operative time, conversion, reoperation, POPF, and blood transfusion	7/9
Zhang 2018 (15)	China	2013-2017	RPD: 20 LPD: 20	RPD:12 LPD: 11	RCS	RPD: 68(50-78) LPD: 64(42-76)	RPD: 24.8(2.5) LPD: 24.0(3.5)	Periampullary tumors	Length of stay, POPF, delayed gastric emptying, reoperation, bile leak, operative time, and blood loss	7/9
Gall 2020 (38)	UK	2017-2019	RPD: 25 LPD: 41	RPD: 16 LPD: 23	RCS	RPD: 60.93(12.52) LPD: 65.18(11.36)	RPD: NA LPD: NA	Benign, or malignant disease	90-day mortality, overall complications, major complications, POPF, reoperation, R0 resection, blood transfusion, conversion, and blood loss	7/9
Klompmaker 2020 (16)	European centers	2012-2017	RPD: 191 LPD: 409	RPD: NA LPD: NA	RCS	RPD: NA LPD: NA	RPD: NA LPD: NA	Solid premalignant tumors or cysts	Major complications, length of stay, POPF, delayed gastric emptying, reoperation, and operative time	6/9
Oosten 2020 (17)	USA	2011-2019	RPD: 90 LPD: 90	RPD: NA LPD: NA	RCS, PSM	RPD: 67(60-73) LPD: 67(58-75)	RPD: 26(23-29) LPD: 25(22-29)	Benign, pre-malignant, or malignant disease	90-day mortality, overall complications, length of stay, POPF, delayed gastric emptying, reoperation, bile leak, blood transfusion, operative time, and blood loss	8/9
Park 2021 (18)	Korea	2016-2020	RPD: 49 LPD: 43	RPD: 26 LPD: 30	RCS	RPD: 66.65(10.97) LPD: 65.70(12.97)	RPD: 23.59(4.28) LPD: 22.73(2.55)	Tumors confined to the pancreatic head or periampullary region	90-day mortality, overall complications, major complications, POPF, delayed gastric emptying, reoperation, bile leak, operative time, and blood loss	8/9
Choi 2022 (19)	Korea	2012-2020	RPD: 50 LPD: 50	RPD: 26 LPD: 29	RCS, PSM	RPD: 60.02(11.97) LPD: 60.42(11.14)	RPD: 23.57(3.18) LPD: 23.99(2.29)	Periampullary tumors	Overall complications, major complications, length of stay, POPF, delayed gastric emptying, reoperation, bile leak, blood transfusion, operative time, and blood loss	8/9
Guo 2022 (20)	China	2016-2020	RPD: 32 LPD: 21	RPD: 21 LPD: 12	RCS	RPD: 53.7(14.4) LPD: 52.1(13.5)	RPD: 21.7(3.0) LPD: 22.6(2.3)	Periampullary tumors	90-day mortality, length of stay, POPF, reoperation, bile leak, conversion, operative time, blood loss, and number of harvested lymph nodes	6/9
Heijde 2022 (21)	European	2019	RPD: 157 LPD: 401	RPD: 67 LPD: 168	PCS	RPD: 62.8(14.7) LPD: 61.8(15.5)	RPD: 26.2(5.0) LPD: 26.7(5.1)	Malignant and benign lesions	90-day mortality, length of stay, POPF, delayed gastric emptying, reoperation, bile leak, conversion, operative time, blood loss	7/9
Jang 2022 (22)	Korea	2012-2020	RPD: 60 LPD: 60	RPD: 28 LPD: 24	RCS, PSM	RPD: 59.5(53.0-64.0) LPD: 58.5(50.0-69.0)	RPD: 23.5(21.6-25.0) LPD: 22.8(20.9-25.0)	Benign or malignant disease (soft pancreas with a small pancreatic duct)	90-day mortality, overall complications, major complications, length of stay, POPF, delayed gastric emptying, reoperation, blood transfusion, conversion, operative time	8/9
Kim 2022 (23)	Korea	Till June 2020	RPD: 74 LPD: 74	RPD: 40 LPD: 42	RCS, PSM	RPD: 57.4(9.5) LPD: 57.8(12.6)	RPD: 23.5(2.7) LPD: 23.5(2.7)	Benign or malignant disease	Overall complications, major complications, length of stay, POPF, delayed gastric emptying, reoperation, R0 resection, bile leak, blood transfusion, conversion, operative time, and number of harvested lymph nodes	6/9
Naffouje 2022 (24)	USA	2004-2017	RPD: 358 LPD: 1074	RPD: 181 LPD: 553	RCS, PSM	RPD: 67.79(10.69) LPD: 67.86(10.31)	RPD: NA LPD: NA	Stage I–III (T1–3 Nany M0) pancreatic adenocarcinoma	90-day mortality, length of stay, R0 resection, conversion, and number of harvested lymph nodes	9/9
Tyutyunnik 2022 (25)	Russia	2007-2015	RPD: 100 LPD: 100	RPD: 43 LPD: 42	RCS	RPD: 62.5(25-84) LPD: 62(34-82)	RPD: 23.1 LPD: 24.2	Malignant and benign tumors of the head of the pancreas and periampullary area	90-day mortality, major complications, length of stay, POPF, delayed gastric emptying, R0 resection, bile leak, blood transfusion, conversion, operative time, and blood loss	7/9
Wach 2022 (26)	USA	2016-2018	RPD: 73 LPD: 73	RPD: NA LPD: NA	RCS, PSM	RPD: NA LPD: NA	RPD: NA LPD: NA	Benign or malignant disease	Overall complications, major complications, length of stay, and conversion	7/9
Zong 2022 (27)	China	2018-2022	RPD: 76 LPD: 114	RPD: 36 LPD: 77	RCS	RPD: 58.2(1.7) LPD: 58.1(1.4)	RPD: NA LPD: NA	Periampullary benign or malignant tumours	Length of stay POPF, delayed gastric emptying, reoperation, bile leak, blood transfusion, conversion, operative time, and blood loss	7/9
Chao 2023 (28)	China	2014-2021	RPD: 75 LPD: 39	RPD: 42 LPD: 15	RCS	RPD: 65.5(58.1-75.5) LPD: 67.1(58.3-74.6)	RPD: 23.8(22.3-27) LPD: 23.7(21.2-25.6)	Periampullary tumors or gastric cancer with pancreatic head invasion	Overall complications, major complications, length of stay, POPF, delayed gastric emptying, reoperation, bile leak, conversion, operative time, blood loss, and number of harvested lymph nodes	7/9
Kalabin 2023 (29)	USA	2010-2018	RPD: 676 LPD: 2677	RPD: 347 LPD: 1390	RCS	RPD: 65.36(64.47-66.25) LPD: 64.97(64.55-65.39)	RPD: NA LPD: NA	Pancreatic adenocarcinoma	90-day mortality, length of stay, R0 resection, and number of harvested lymph nodes	7/9
Khachfe 2023 (30)	USA	2014-2019	RPD: 885 LPD: 655	RPD: 462 LPD: 347	RCS	RPD: 67(59-73) LPD: 65(5772)	RPD: 27.1(23.7-31.1) LPD: 26.95(23.7-30.4)	Benign or malignant disease	Overall complications, POPF, delayed gastric emptying, blood transfusion, conversion, and operative time	7/9
Lee 2023 (31)	Korea	2015-2019	RPD: 21 LPD: 60	RPD: 10 LPD: 28	RCS	RPD: 57.7(11.6) LPD: 68.2(8.5)	RPD: 23.3(1.6) LPD: 23.6(2.3)	Distal bile duct cancer	Major complications, length of stay, POPF, R0 resection, blood transfusion, and blood loss	7/9
Uijterwijk 2023 (32)	8 centers (6 in Europe, 1 in Australia, and 1 in Asia)	2010-2021	RPD: 37 LPD: 53	RPD: NA LPD: NA	RCS	RPD: NA LPD: NA	RPD: NA LPD: NA	Distal cholangiocarcinoma	Overall complications, length of stay, POPF, delayed gastric emptying, bile leak, blood transfusion, operative time, blood loss, and number of harvested lymph nodes	6/9
Wei 2023 (33)	China	2014-2021	RPD: 78 LPD: 45	RPD: NA LPD: NA	PCS	RPD: NA LPD: NA	RPD: NA LPD: NA	NA	Major complications, POPF, and delayed gastric emptying	6/9
Zhang 2023 (1)	China	2015-2022	RPD: 1006 LPD: 1006	RPD: 612 LPD: 622	RCS, PSM	RPD: 60.5(52.0-67.0) LPD: 61.0(52.0-67.0)	RPD: 23.4(21.3-25.2) LPD: 23.1(20.9-25.5)	Benign, premalignant, or resectable malignant or borderline resectable tumors of the pancreatic and periampullary region	90-day mortality, length of stay, major complications, POPF, delayed gastric emptying, reoperation, R0 resection, bile leak, blood transfusion, conversion, operative time, blood loss, and number of harvested lymph nodes	9/9
Dai 2024 (35)	China	2016-2023	RPD: 47 LPD: 54	RPD: 27 LPD: 32	RCS	RPD: 59.8(10.6) LPD: 60.5(12.2)	RPD: 22.44(3.31) LPD: 23.59(4.17)	Pancreatic Cancer	90-day mortality, major complications, length of stay, POPF, delayed gastric emptying, reoperation, bile leak, conversion, operative time, blood loss, and number of harvested lymph nodes	8/9
Kang 2024 (36)	Korea	2015-2020	RPD: 332 LPD: 178	RPD: 185 LPD: 94	RCS	RPD: 63.6(12.1) LPD: 67.5(11.8)	RPD: 23.5(2.6) LPD: 24.3(2.9)	Benign or malignant periampullary tumors	Major complications, length of stay, POPF, operative time, conversion, and blood loss	7/9
Kuriyama 2024 (37)	Japan	2020-2024	RPD: 41 LPD: 16	RPD: 23 LPD: 13	RCS	RPD: 65(39-84) LPD: 72(44-91)	RPD: 22.9(15.3-31.9) LPD: 22.7(16.9-31.1)	NA	Major complications, length of stay, POPF, delayed gastric emptying, reoperation, bile leak, operative time, and blood loss	7/9
Emmen 2024 (53)	50 centers in 12 European countries	2009-2020	RPD: 812 LPD: 812	RPD: 416 LPD: 428	RCS, PSM	RPD: 76(58-74) LPD: 66(57-73)	RPD: 24.7(22.5-27.7) LPD: 24.6(22.1-27.6)	NA	Major complications, length of stay, POPF, delayed gastric emptying, reoperation, bile leak, conversion, operative time, blood loss, and number of harvested lymph nodes	8/9
Farah 2024 (4)	USA	2014-2021	RPD: 175 LPD: 100	RPD: NA LPD: NA	RCS	RPD: NA LPD: NA	RPD: NA LPD: NA	Pancreatic cancer	Overall complications, major complications, POPF, delayed gastric emptying, blood transfusion, and conversion	7/9
Wehrle 2024 (34)	USA	2010-2020	RPD: 625 LPD: 625	RPD: 323 LPD: 332	RCS, PSM	RPD: 6.5(10.4) LPD: 65.6(10.1)	RPD: NA LPD: NA	Pancreatic cancer	90-day mortality, length of stay, R0 resection, conversion, and number of harvested lymph nodes	9/9

LPD, laparoscopic pancreaticoduodenectomy; NA, not available; PCS, prospective retrospective cohort study; POPF, postoperative pancreatic fistula; PSM, propensity score matching; RCS, retrospective cohort study; RPD, robotic pancreaticoduodenectomy.

### Meta-analysis

#### 90-day mortality

**Figure 2:**
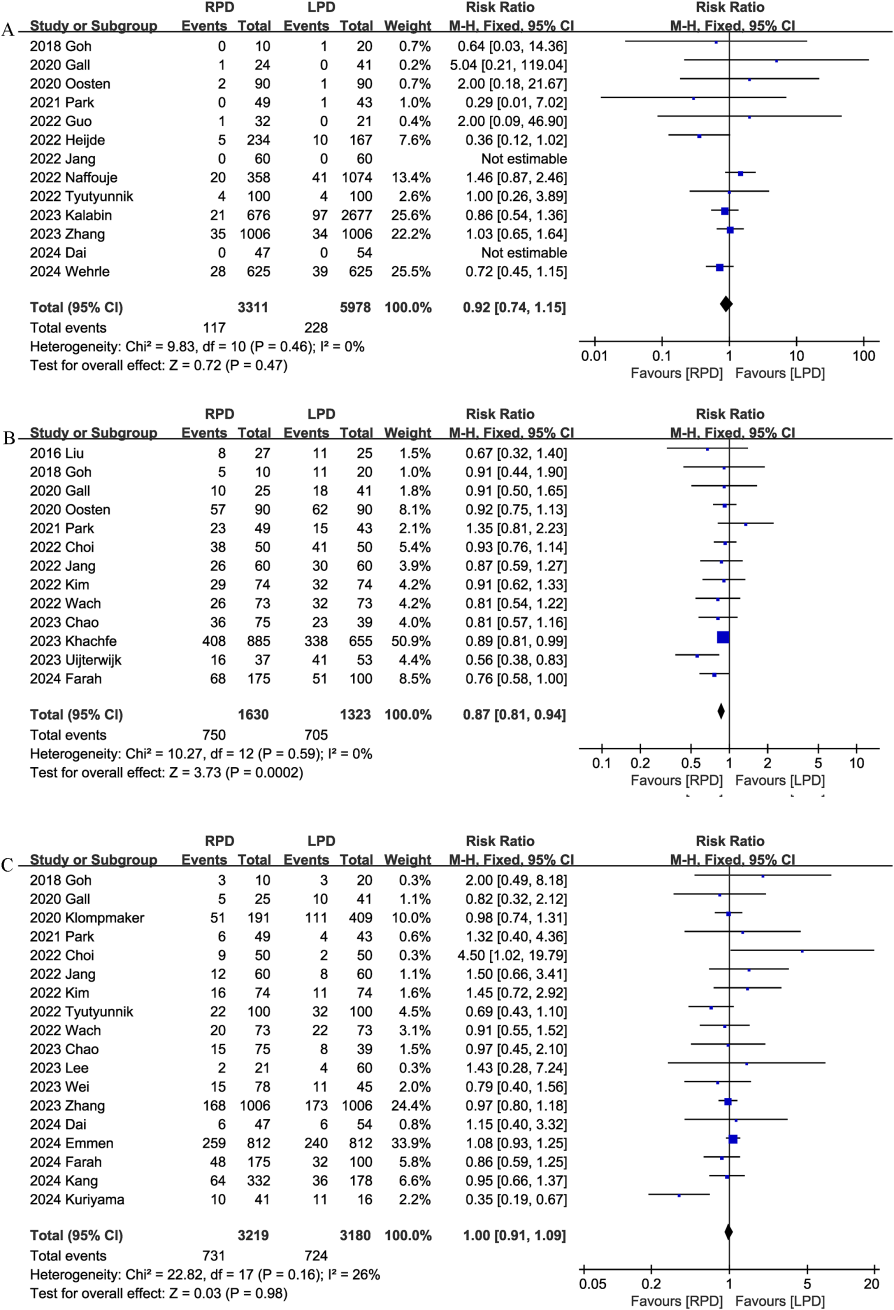
Comparison of primary outcomes between the two groups. **(A)** 90-day mortality, **(B)** overall complications, and **(C)** major complications.

Thirteen studies reported data on 90-day mortality. The combined results of the 13 studies showed that there was no significant difference between the RPD group and the LPD group regarding this outcome with low heterogeneity (RR 0.92, 95% CI 0.74, 1.15; Heterogeneity: I^2^ = 0%, P = 0.46) (**Figure 2A**).

#### Overall complications

Thirteen studies assessed overall complications. The pooled results suggested that RPD significantly reduced the overall complication rates (RR 0.87, 95% CI 0.81, 0.94, P = 0.0002), with low heterogeneity (I^2^ = 0%, P = 0.59) (**Figure 2B**).

#### Major complications

Combined data from 18 studies showed that the rates of major complications (Clavien–Dindo ≥ 3) were comparable between the RPD and LPD groups (RR 1.00, 95% CI 0.91, 1.09; Heterogeneity: I^2^ = 26%, P = 0.16) (**Figure 2C**).

#### Length of stay

**Figure 3:**
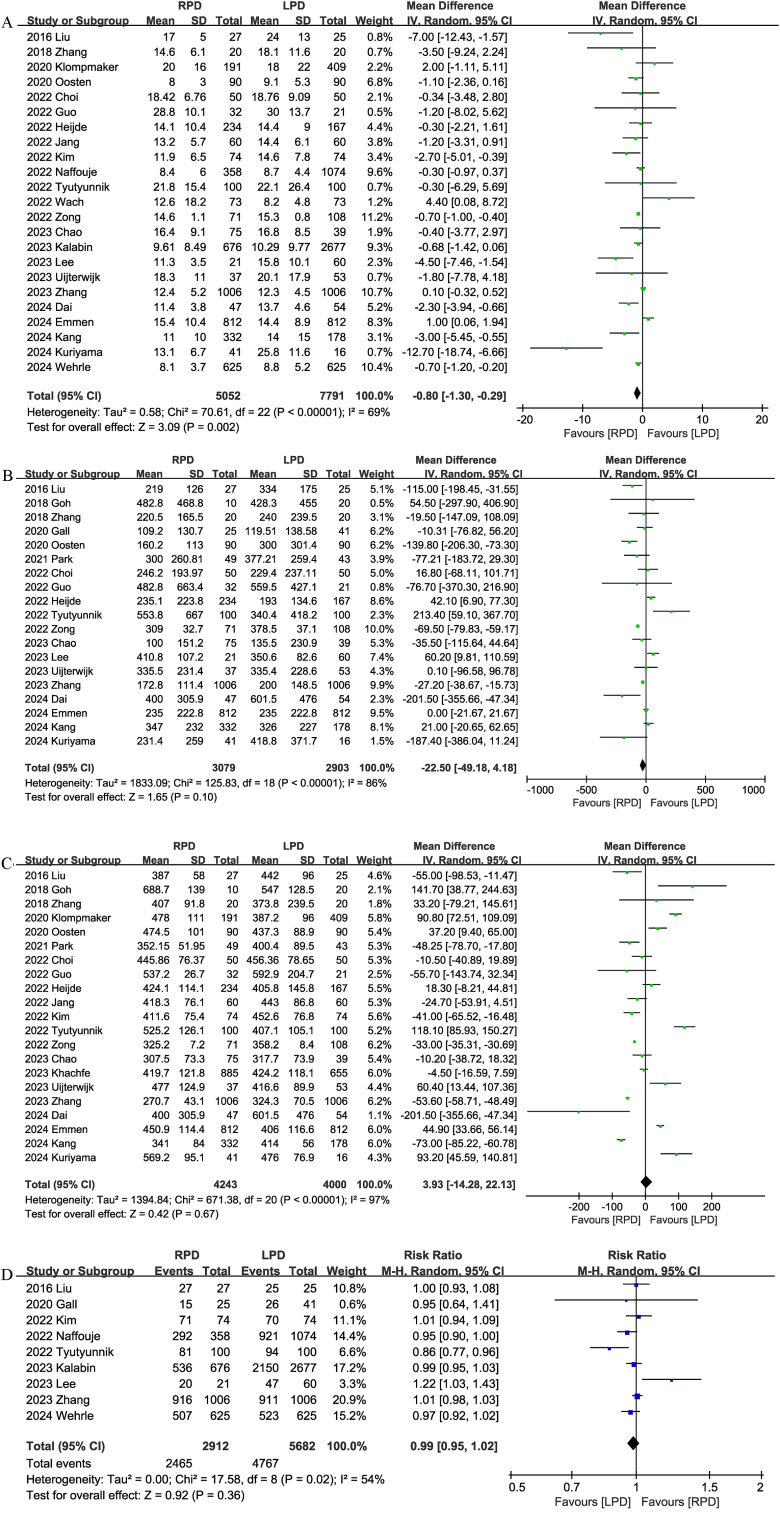
Comparison of secondary outcomes between the two groups. **(A)** length of stay, **(B)** intraoperative blood loss, **(C)** operative time, and **(D)** R0 resection.

The length of the hospital stay was reported in 23 studies. According to the results of this meta-analysis, RPD significantly reduced the length of the hospital stay as compared with the LPD group (MD, -0.80 days; 95% CI, -1.30, -0.29, P = 0.002) (**Figure 3A**).

#### Blood loss

Nineteen studies provided information on intraoperative blood loss. The combined results showed that the RPD group has similar intraoperative blood loss as compared with the LPD group (MD, -22.50 mL; 95% CI, -49.18, 4.18, P = 0.10; I^2^ = 86%) (**Figure 3B**).

#### Operation time

The operation time was reported in 21 trials. The combined results showed that the RPD group has similar operation time as compared with the LPD group (MD, 3.93 mins; 95% CI, -14.28, 22.13, P = 0.67) (**Figure 3C**).

#### R0 resection

R0 resection was reported in 9 studies, and the combined effect size suggested that the R0 resection rates were comparable between the two groups (RR 0.99, 95% CI 0.95, 1.02, P = 0.36; I^2^ = 54%) (**Figure 3D**).

#### Number of lymph nodes harvested

**Figure 4:**
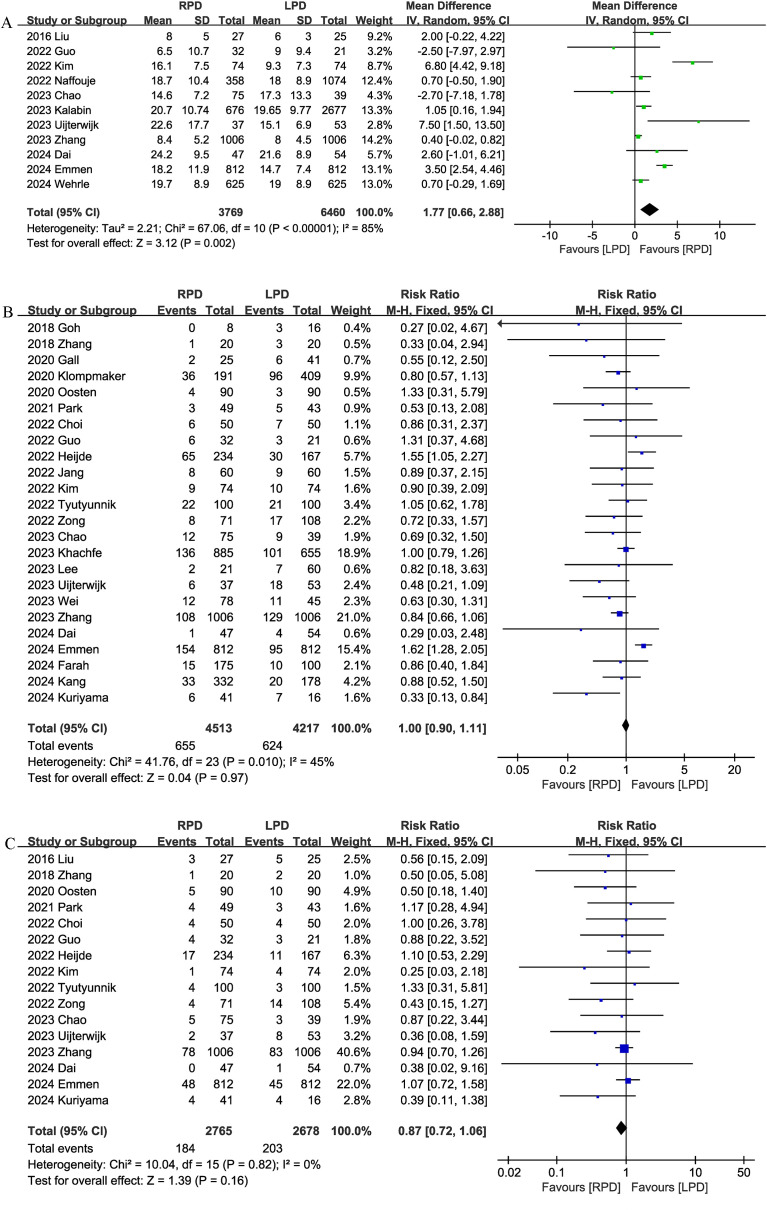
Comparison of secondary outcomes between the two groups. **(A)** number of lymph nodes harvested, **(B)** postoperative pancreatic fistula, and **(C)** bile leak.

Eleven trials reported the number of lymph nodes harvested. Compared with LPD, RPD significantly increased the number of lymph nodes harvested (MD, 1.77; 95% CI, 0.66, 2.88, P = 0.002; I^2^ = 85%) (**Figure 4A**).

#### Postoperative pancreatic fistula

Twenty-four studies evaluated the POPF. There was no significant difference in the incidence of POPF (RR 1.00, 95% CI 0.90, 1.11, P = 0.97) (**Figure 4B**) between the RPD and LPD groups.

#### Bile leak

Sixteen studies reported bile leaks. No significant differences were observed between the two groups (RR 0.87, 95% CI 0.72, 1.06, P = 0.16), and heterogeneity was low (I^2^ = 0%, P = 0.82) (**Figure 4C**).

#### Conversion rate

**Figure 5:**
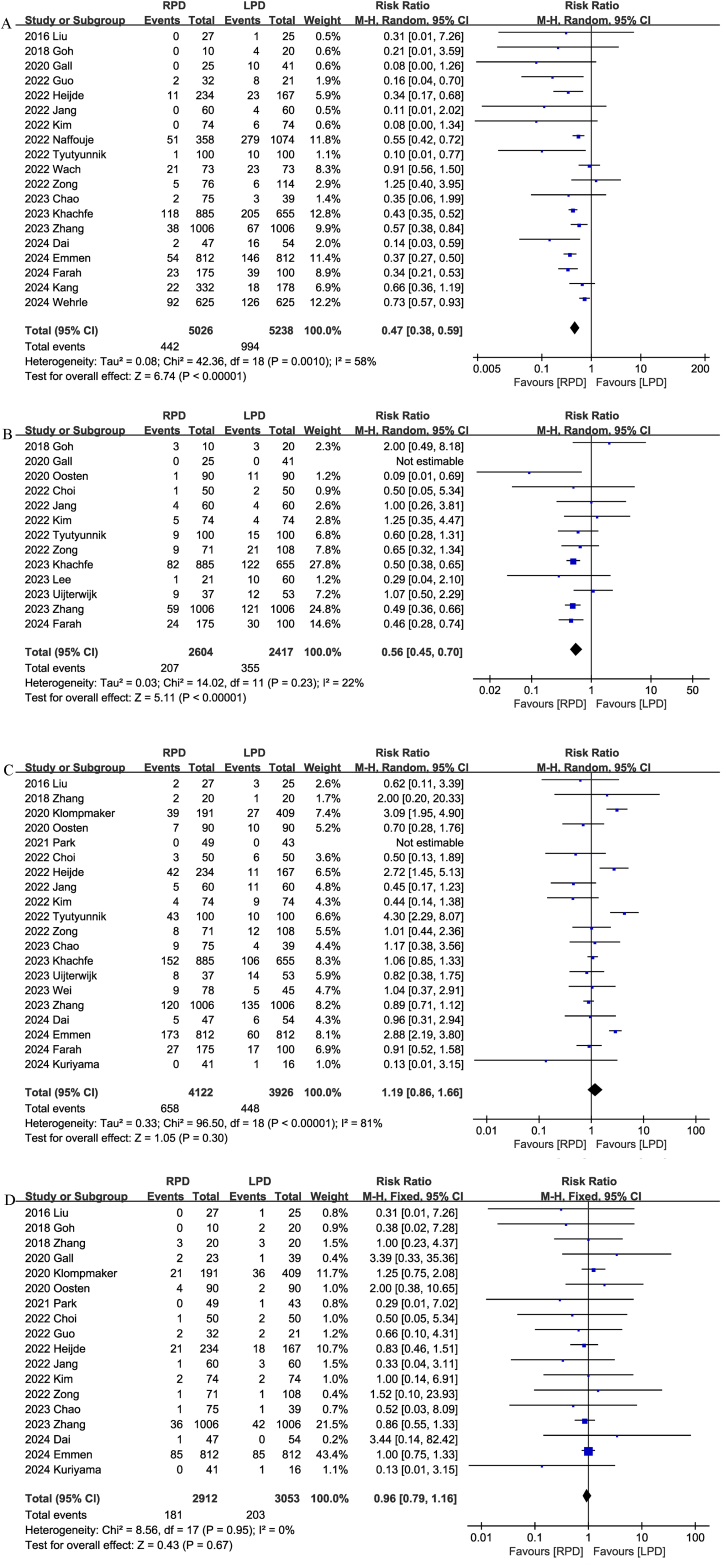
Comparison of secondary outcomes between the two groups. **(A)** Conversion rate, **(B)** blood transfusion, **(C)** delayed gastric emptying, and **(D)** reoperation.

Conversion rate was evaluated in 19 studies, and the pooled results showed that RPD had lower conversion rate than LPD (RR 0.47, 95% CI 0.38, 0.59; heterogeneity: I^2^ = 58%, P = 0.0010) (**Figure 5A**).

#### Blood transfusion

Thirteen studies compared blood transfusion rates between the RPD and LPD groups. The combined results showed that RPD was effective in reducing the blood transfusion rate (RR 0.56, 95% CI 0.45, 0.70, P<0.00001) (**Figure 5B**).

#### Delayed gastric emptying

Delayed gastric emptying was reported in 20 studies, and there was no significant difference in the incidence of delayed gastric emptying (RR 1.19, 95% CI 0.86, 1.66, P = 0.30) (**Figure 5C**) between the two groups.

#### Reoperation

Eighteen trials reported the reoperation rates. There were no significant differences between the two groups, and heterogeneity was low (RR 0.96, 95% CI 0.79, 1.16; Heterogeneity: I^2^ = 0%, P = 0.95; **Figure 5D**).

### Sensitivity analysis

**Figure 6:**
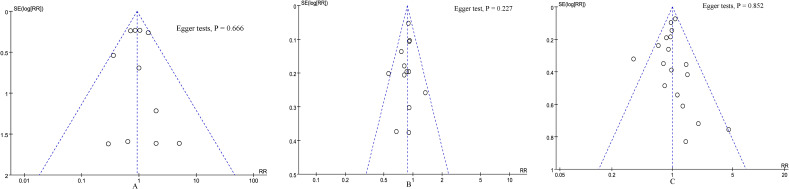
Funnel plot of primary outcomes. **(A)** 90-day mortality, **(B)** overall complications, and **(C)** major complications.

According to the funnel plots (**Figure 6**) and Egger tests, and no significant publication bias was observed for 90-day mortality, overall complications, and major complications. Sensitivity analysis showed that no single study affected the overall effect size of the length of stay, blood transfusion, conversion rate, 90-day mortality, overall complications, major complications, reoperation, bile leak, operation time, delayed gastric emptying, POPF, number of lymph nodes harvested, blood loss, or R0 resection. Excluding these studies with no events in one or both groups did not change the total effect size of blood transfusion, conversion rate, 90-day mortality, reoperation, bile leak, delayed gastric emptying, and POPF.

## Discussion

In recent years, minimally invasive surgery has been widely used in pancreatic surgery. However, whether RPD is superior to LPD remains controversial. Although two previous meta-analyses (39, 40) were conducted, they included only six and nine studies, respectively, limiting the reliability of their conclusions. In comparison, our study included 29 studies, including data from 15137 patients. Our meta-analysis showed that compared with traditional LPD, RPD effectively reduced postoperative complications, blood transfusion, and conversion rates, shortened hospital stay, and increased the number of lymph nodes harvested. In addition, there were no significant differences in postoperative mortality, reoperation rates, operation time, intraoperative blood loss, and R0 resection rates between the two groups. Our results have important clinical value as we provide evidence that RPD is not inferior to LPD in the short term and can provide potential benefits. These results may help pancreatic surgeons in their choice of surgical approaches.

Postoperative complications are associated with a poorer long-term prognosis (3). Cho et al. (41) analyzed 200 patients with periampullary cancer who underwent pancreatoduodenectomy and showed that 3-year overall survival and disease-free survival were significantly lower in patients with postoperative complications (31.0% and 22.3%, respectively) than in patients without postoperative complications (49.0% and 40.0%, respectively). The high complication rate after PD is troubling pancreatic surgeons, and minimally invasive surgery may be a potential strategy to improve the postoperative morbidity of PD. Surgeons’ enthusiasm for LPD waned due to the high mortality rates reported in the LEOPARD-2 trial (42). In addition, subsequent meta-analyses (43) based on RCTs have also failed to demonstrate the benefit of LPD in terms of postoperative complications, leading to increasing hopes for RPD. Our results showed that RPD significantly reduced the incidence of postoperative complications compared with LPD. Given the impact of postoperative complications on long-term survival, lower postoperative complications may have potential benefits for patients’ long-term outcomes. In 2020, Kamarajah et al. (39) conducted a meta-analysis of six non-RCTs, involving 3,462 patients. Their results indicated that there was no significant difference in the incidence of postoperative complications and POPF compared with LPD and RPD. In 2022, Ouyang et al. (40) conducted an updated meta-analysis, and their study included nine retrospective studies. The results of the meta-analysis indicated that there were no significant differences between RPD and LPD in terms of total postoperative complications, major complications, POPF, delayed gastric emptying, and reoperation. Furthermore, the meta-analysis by Armengor-Garcia et al. (44) included 17 studies involving a total of 5,483 patients. The results indicated that compared with LPD, RPD did not significantly reduce postoperative hemorrhage, delayed gastric emptying, mortality, or readmission rates. However, Armengol-Garcia et al. did not evaluate the data of total postoperative complications and major complications. A meta-analysis by Tang et al. (45), which included 17 studies and 9,417 subjects, indicated that RPD could significantly reduce postoperative complications. Compared with previous studies, our meta-analysis has the following innovations. On the one hand, the number of studies and sample sizes included in the previously published meta-analyses were limited, which affected the statistical power and failed to draw convincing conclusions. In contrast, we included a larger number of studies (29 studies) and a larger sample size (15137 subjects), making our results more reliable. On the other hand, the population we included was broader, including patients with non-ampullary tumors, which made our conclusion more universal. In addition, Conversion to open is associated with an increased risk of postoperative complications (39). Our summarized results suggest that the conversion rates in the RPD group is significantly lower than that in the LPD group. Similarly, several previously published studies have observed the benefit of robotic surgery in reducing conversion rates in a variety of procedures (46–48). POPF is the most common and destructive complication after PD surgery, with an incidence of up to 20% (49). POPF is classified by the International Pancreatic Surgery Research Group (ISGPS) into clinically relevant POPF (Grade B and C) and biochemical POPF (Grade A) (50). Our study showed no significant difference between RPD and LPD in the incidence of clinically relevant POPF. This is consistent with the results of two previous meta-analyses (39, 40).

Increased intraoperative blood loss is significantly associated with poor prognosis in PD, and reducing intraoperative blood loss is helpful to improve perioperative outcomes (51). One of the advantages of minimally invasive surgery is that it is less invasive and less bleeding during the operation (52). Compared to LPD, RPD has a wider field of view, fewer tremors, and can perform detailed anatomy with less surgical trauma (40, 52). These advantages may lead to benefits in reducing intraoperative blood loss. Our findings showed that RPD significantly reduced the blood transfusion rate.

Some researchers are concerned that robotic surgery may prolong the operation time because of the additional time required to assemble the equipment (52, 53). However, a recent study (3) found that when the surgical team goes beyond the learning curve and gains enough experience, the surgical time for RPD is significantly reduced. A previous meta-analysis by Kamarajah et al. (39) found that RPD did not extend surgery time compared to LPD. The results of this study also indicated that the operation time was comparable between the RPD group and the LPD group. In addition, previous evidence has shown that robot-assisted gastrointestinal surgery can improve gastrointestinal function recovery and shorten hospital stays compared to laparoscopic surgery (54). In PD surgery, we also demonstrated the benefit of RPD in reducing the length of hospital stay.

Complete tumor resection and appropriate lymph node dissection are the keys of PD. R0 resection is an important predictor of long-term survival (49). A previous meta-analysis (49) showed no significant difference in R0 resection rates between different surgical approaches (open PD, LPD, and RPD). This is similar to the results of this study. Obtaining a sufficient number of lymph nodes is critical for accurate assessment of lymph node status, and the number of lymph nodes obtained is significantly associated with accurate staging and long-term patient survival (55). Our study showed that RPD significantly increased the number of lymph nodes acquired compared with LPD. This may be due to the robotic platform’s ability to provide enlarged 3D images that eliminate arm tremors and aid in precise lymph node dissection (40).

The high cost may be a factor limiting the further adoption of RPD. Due to the lack of data related to hospitalization costs in the included studies, we did not assess the difference in total costs between RPD and LPD. In fact, the increase in the cost of robotic surgery is mainly due to the installation and maintenance of the equipment (30). For example, in other areas such as hepatectomy and distal pancreatectomy, some studies have found that the surgical cost of robotic surgery is higher than laparoscopic surgery, while the hospital cost of robotic surgery is lower than laparoscopic surgery (56, 57). With the development of technology and the popularity of robotic surgery, the equipment cost of RPD is expected to decrease. In addition, the benefits of robotic surgery (lower postoperative complications and shorter hospital stays) may further reduce hospital costs. Therefore, the economic benefits of RPD deserve further evaluation in future studies.

This study has the following strengths. On the one hand, we conducted an extensive literature search, incorporating all the evidence currently available. On the other hand, we confirmed the robustness of the main results through sensitivity analysis.

There are some limitations to this study. First, most of the studies included in this meta-analysis are retrospective studies and lack RCTs. Second, high heterogeneity was found in some outcome measures (length of hospital stay, number of lymph nodes harvested, and operation time), which hindered accurate estimation of outcomes. The included studies originate from different countries, which may introduce variability in surgical standards, healthcare infrastructure, and patient management protocols. These differences may be the sources of heterogeneity. However, the sensitivity analysis still confirmed the stability of our main results. Furthermore, most of the included studies originated from high-volume centers. The availability of robotic surgery is limited in some developing countries. Considering the differences among regions, the conclusions of our research may not be directly generalized to some low-volume units. These low-volume centers need to undergo further training with RPD and go through the learning curve in order to bring out the true benefits of RPD. Among the 29 studies we included, 9 studies adopted the PSM design, while the remaining studies did not. The failure to adopt the PSM design may lead to differences in some preoperative basic characteristics (such as age, gender and weight), and these factors may have an impact on the results of the study. In the future, well-designed RCTs are needed to further balance the differences between the experimental group and the control group to verify the benefits of RPD. Finally, although our meta-analysis suggests that RPD is no less safe and effective than LPD in the perioperative period, few studies have evaluated the difference in long-term oncology outcomes between RPD and LPD. Given the potential benefits of RPD, future well-designed studies investigating the long-term oncology prognosis of RPD are warranted.

In conclusion, this meta-analysis suggests that compared with LPD, RPD can significantly reduce postoperative complications, blood transfusion, conversion, and hospital stay, and increase the number of lymph nodes harvested. In addition, there were no significant differences in mortality, reoperation rates and R0 resection rates between the two procedures.

## Data Availability

The original contributions presented in the study are included in the article/supplementary material. Further inquiries can be directed to the corresponding authors.
